# Influence of Poly (benzyl oleate-co-maleic anhydride) Pour Point Depressant with Di-Stearyl Amine on Waxy Crude Oil

**DOI:** 10.3390/polym15020306

**Published:** 2023-01-06

**Authors:** Marwa R. Elkatory, Mohamed A. Hassaan, Emad A. Soliman, Violeta-Carolina Niculescu, Maria Simona Raboaca, Ahmed El Nemr

**Affiliations:** 1 Polymer Materials Research Department, Advanced Technology and New Materials Research Institute, SRTA-City, Alexandria 21934, Egypt; 2 Environment Division, Marine Pollution Department, National Institute of Oceanography and Fisheries (NIOF), Alexandria 21556, Egypt; 3 National Research and Development Institute for Cryogenic and Isotopic Technologies—ICSI Ramnicu Valcea, 4th Uzinei Street, 240050 Valcea, Romania; 4 Doctoral School, University Politehnica of Bucharest, Splaiul Independentei Street No. 313, 060042 Bucharest, Romania

**Keywords:** pour point depressants, flow improvers, copolymerization, fatty acids, crystallization of wax, waxy crude oil

## Abstract

An important problem for the oil industry is the deposition of paraffin on pipelines during the transit of crude oil and restart processes at low temperature. In this regard, the need for suitable methods of wax deposition has attracted substantial attention. Therefore, pour point depressants (PPDs) are considered a critical processing aid to modify the paraffin crystallization and improve the flow of waxy crude oil. The effect of pendants in comb-type copolymers on the ability of crude oil to flow in the cold is examined in the current study. Such PPDs were first created by the free radical polymerization of maleic anhydride with benzyl oleate to create the poly (benzyl oleate-co-maleic anhydride). The resultant copolymer was then aminated with alkyl amine (stearyl amine) (C_18_H_39_N) to form pendant alkyl amine chains. The esterified copolymers were structurally characterized by Fourier Transform Infrared, X-ray diffraction spectral analysis, and scanning electron microscopy. Moreover, the potential interactions between PPD and waxes were investigated by using differential scanning calorimetry, X-ray diffraction, and light microscopy. The obtained PPDs, which are effective at a dose of 2000 ppm, were able to reduce the pour point by up to 3 °C. The viscosity and yield stress of the petroleum waxy crude oil were revealed by rheometer.

## 1. Introduction

As a fossil fuel type, waxy crude oil is widespread throughout the world. N-paraffin with carbon numbers between 18 and 65 comprise most of its content [1]. When oil is extracted from offshore areas where temperatures may fall below 4 °C, lower than the wax appearance temperature (WAT) of crude, a “house-of-cards” structure of orthorhombic wax-crystals forms, resulting in a three-dimensional network. This might cause paraffin to accumulate on the pipeline’s surface [2]. The deposition of paraffin in pipelines when oil is being transported raises the cost of pumping and reduces the efficiency of the system. The oil industry suffers billions of dollars in damages every year as a result of partial or complete line blockages [3,4]. Crude oil flowability is affected by a wide range of variables, including its chemical components, temperature, and both the present and prior temperatures [1,5]. Academic and industrial wax deposition issues have been addressed using a variety of techniques. One or a combination of chemical, mechanical, and thermal remediation techniques can be used to minimize or prevent wax deposition in crude oil production systems. The employment of mechanical and thermal remediation methods, however, has become economically unfeasible with the development of extremely deep production, offshore drilling, and ocean floor completions. As a result, chemical additives serving as wax deposition inhibitors are being used more frequently. The thermal methods include heat retention, active heating such as thermal insulations, bottom hole heaters, hot oil circulation, and steam circulation. The mechanical removal approach entails pigging in pipes at a predetermined frequency and running scrappers in the borehole. Pour point depressants (PPD), crystal modifiers, dispersants, and solvents are a few examples of chemical inhibitors [5]. Chemicals are added to waxy oil to prevent wax deposition on the pipe’s surface. The oil industry has made extensive use of chemicals such as wax-crystal modifiers (paraffin inhibitors), solvents, and dispersants [6,7,8]. Because of their potential to modify wax crystals, wax-crystal modifiers can minimise crystallization and promote flowability by altering the crystal structure of waxes [8]. Mechanisms for wax-crystal alteration that have been universally recognized so far include adsorption, co-crystallization, dissolution, and nucleation [9]. The problem of paraffin deposition is said to be greatly alleviated by the pour point depressant’s alkyl portion being similar to that of paraffin [1,10]. Non-polar alkyl components (paraffins) and polar components are found in the typical structure of wax modifiers. Paraffin molecules can be co-crystallized with aromatic molecules, although the polar portion prevents crystals from growing [11]. PPDs, crude oil composition, transportation conditions, and other factors all have a role in crude oil flowability [12,13,14]. Additives such as ethylene–vinyl acetate copolymers (EVA) [15], alkyl acrylates [16], and polymers of maleic acid alkyl amide-olefin-styrene amide have all been employed as PPDs in the last few years. It is possible to buy EVA and acrylic polymers that have comb-like structures on the market. Pour point was reduced and rheological properties were improved when long chain ionic liquids such as bis(trifluoromethanesulfonyl) imide, 1-dodecyl-3-methylimidazolium 1-tetradecyl-3-methylimidazolium bis(trifluoromethanesulfonyl) imide, and poly(n-alkyl recinoleate-co-N-hexadecylmaleimide) were used as PPDs, according to the research of [17,18,19]. Al-Sabagh et al. [20] investigated whether the copolymer with aliphatic side chain styrene maleic anhydride co-amide (SMACA) exhibited the maximum pour point depression (ΔPP = 21 °C at 1500 ppm). This work aimed to study the performance of the comb-shaped copolymers of benzyl oleate (biobased) as a start material copolymerized with maleic anhydride, and amidation with alkyl amines, as PPDs were assessed in the current work. Rheological, differential scanning calorimetry (DSC), X-ray diffraction (XRD), and light microscopy (LM) techniques were used to evaluate Egyptian waxy crude oil in order to investigate the yield value and wax-crystal modifications.

## 2. Materials and Methods

### 2.1. Materials

Maleic anhydride, oleic acid, benzyl alcohol, octadecylamine, N-butyl titanate, toluene, and p-toluene were purchased from Sigma-Aldrich (St. Louis, MO, USA).

### 2.2. Petroleum Crude Oil

The waxy crude oil was obtained from the Alamin field located in Egypt’s Western Desert. Like most of the producing fields in the Western Desert, Alamin field produces a paraffinic crude with a relatively high pour point. Alamin crude oil is produced by Amerya Petroleum Refining Co. (APRC) (Alexandria, Egypt). Its physicochemical properties are listed in Table 1.

### 2.3. Esterification of Oleic Acid and Benzyl Alcohol

Fatty acid benzyl esters were prepared by esterification of the corresponding oleic acids and benzyl alcohol. N-butyl titanate (250 μL), fatty acid (25 g), and benzyl alcohol (30 g) were dissolved in toluene (40 mL) and refluxed for 10 h to ensure the complete conversion of fatty acid. The water produced during esterification was removed by the azeotrope of toluene and water. After reaction, toluene and excess benzyl alcohol were removed under vacuum [19].

### 2.4. Preparation of Poly (benzyl oleate-co-succinic anhydride) Copolymer (PBOCOSA)

Using benzoyl peroxide as the initiator (1% *w*/*w*), the pre-prepared benzyl oleate was copolymerized with maleic anhydride in toluene at molar ratios of 1:2 and 1:1, respectively. Then, 20 h of continuous stirring at 90 °C was used to complete the polymerization. Petroleum ether 40/60 was used to precipitate the copolymer, and the same mixture was also used to filter and wash the copolymer. At 60 °C, the product was dried [5].

### 2.5. Preparation of Poly (benzyl oleate-co-distearyl amine) (PBOCODSA)

**Scheme 1:**
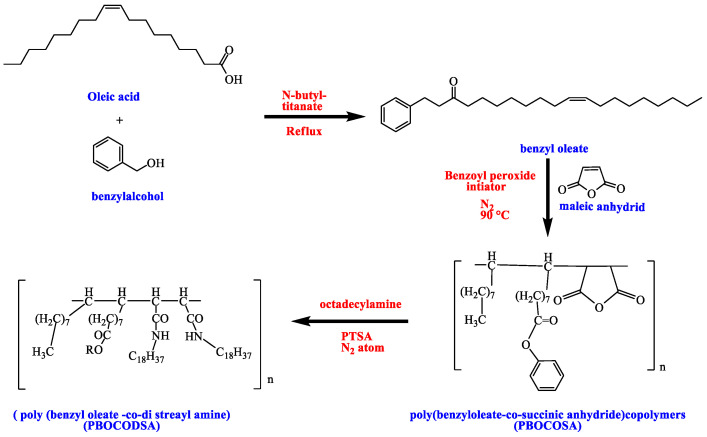
Schematic illustration of preparation of PBOCODSA.

The prepared PBOCOSA was allowed to reflux individually with octadecyl amine (*Amidation of PBOCOSA)* in 1:2 molar ratio for 8 h, in the presence of p-toluene sulfonic acid (0.1%) as a catalyst and toluene as a solvent. After the collection of the calculated volume of water, the products were precipitated in excess methanol and then washed several times, filtered, and dried overnight [20]. The amidation reactions of PBOCOSA copolymer to produce PBOCODSA copolymer are shown in Scheme 1.

### 2.6. Characterization of Structural Properties of Polymeric Additives

The structure of the prepared PBOCOSA and PBOCODSA was confirmed by using FTIR Shimadzu 8400S spectrometer (Shimadzu, Japan). The morphological features microstructures of copolymers and their esterified forms and esters were examined by a Jeol 6360LA SEM (JEOL Ltd., Tokyo, Japan), working at an acceleration voltage of 15 kV [2]. XRD for PBOCOSA and PBOCODSA were obtained using a Shimadzu XRD-7000 diffractometer (Shimadzu Co. Ltd., Kyoto, Japan) (30 kV, 30 mA) with Cu Kα, Ni-filtered radiation, and the sample scanned at 2θ from 4–100° at a rate of 4°/min. The thermal stability of PBOCODSA (∼6 mg) was performed using a thermogravimetric analyser (TGA) device (Shimadzu TGA-50/50H, Japan) at a temperature from ambient—800 °C with a heating rate of 10 °C /min under nitrogen flow (20 mL/min).

### 2.7. Performance Evaluation of the Synthesized Polymeric Additives

#### 2.7.1. Pour Point (PP) Measurements (ASTM D97)

Solutions of the synthesized PBOCOSA and PBOCODSA in xylene (containing 20% active material) were prepared at various concentrations (500, 1000, 2000, and 3000 ppm). To ensure the complete breakdown of the paraffin content, the solutions were injected into the crude oil at 60 °C, swirled for homogenization, and then submitted to the pour point test in accordance with ASTM D97 standard procedure without warming [21]. The outcomes were shown as a decrease in the pour point of pure crude oil, which was computed using the equation:

Pour point reduction (ΔP) = PP_pure_ − PP_additive_ (1)

where PP_pure_ is the pour point of the pure crude oil and PP_additive_ is the pour point of the crude oil containing additives [22].

#### 2.7.2. Rheological Measurements

Using a Brookfield DV-II programmable viscometer, the effectiveness of PBOCODSA in improving the low-temperature fluidability of waxy crude oil was assessed by comparing the rheological characteristics of samples of untreated crude oil and counterpart samples treated with various concentrations of the pre-prepared polymeric additives (500, 1000, and 2000 ppm). The experiment began when the additives were combined with crude oil at the specified concentration and temperature (60 °C). In the meantime, the viscometer cup was preheated to the same temperature and filled with 25 mL of the sample, before the temperature was lowered to a constant level where the measurements could be made at a low shear rate of 0.024 s^−1^ (dynamic cooling). For evaluation, shearing was maintained for an additional 15 min at the test temperature. For the examined samples, the link between shear stress and shear rate was noted. The equations that can be used to compute the shear rate, shear stress, and viscosity are presented below [23]:

Shear rate (D) = M × n (s^−1^) (2)

M is the shear-rate factor and n is the actual test speed. The actual test speed is calculated as follows:

n = set test speed/reduction factor (R)(3)

Shear stress (τ) = A × S (Pa)(4)

A is the shear-stress factor and S is the measuring value (torque).

Apparent viscosity (η) = (G × S)/n (m Pa·s)(5)

G is an instrument factor [23].

#### 2.7.3. Crystallization Behaviour and Morphological Features Examination

##### DSC

DSC analyses were performed with a Shimadzu DSC-60A calorimeter (Shimadzu Co. Ltd., Kyoto, Japan). After the waxy oil was heated to 70 °C to dissolve the wax completely, approximately 1 mg of waxy oil was placed in a DSC sample pan measured using equipment with a data processor

DSC was first calibrated with indium and purged with nitrogen gas atmosphere. The samples were allowed to cool to −25 °C at a constant cooling rate of 10 °C/min and scanned from −25 °C to 70 °C at a constant heating rate of 10 °C/min [24].

##### XRD

A graphite monochromator was selected (40 kV, 30 mA) with Cu Kα radiation and the sample was scanned at a rate of 4 °C/min in the range of 4–70° in 2θ, with step size of 0.02° in 2θ [24,25].

##### Photomicrography

Using an Olympus BH2-UMA microscope coupled with a Canon photometric high-resolution automatic digital camera, the morphological characteristics of the paraffin wax crystals present in the untreated and crude oil sample treated with the synthesized additives at different concentrations were recorded. A drop of crude oil was deposited on a slide and the sample was examined while cooling at a rate of 10 °C/min [2,5].

## 3. Results and Discussion

### 3.1. Structural Characterization of Copolymer and Its Derived Structure

#### 3.1.1. FTIR

**Figure 1:**
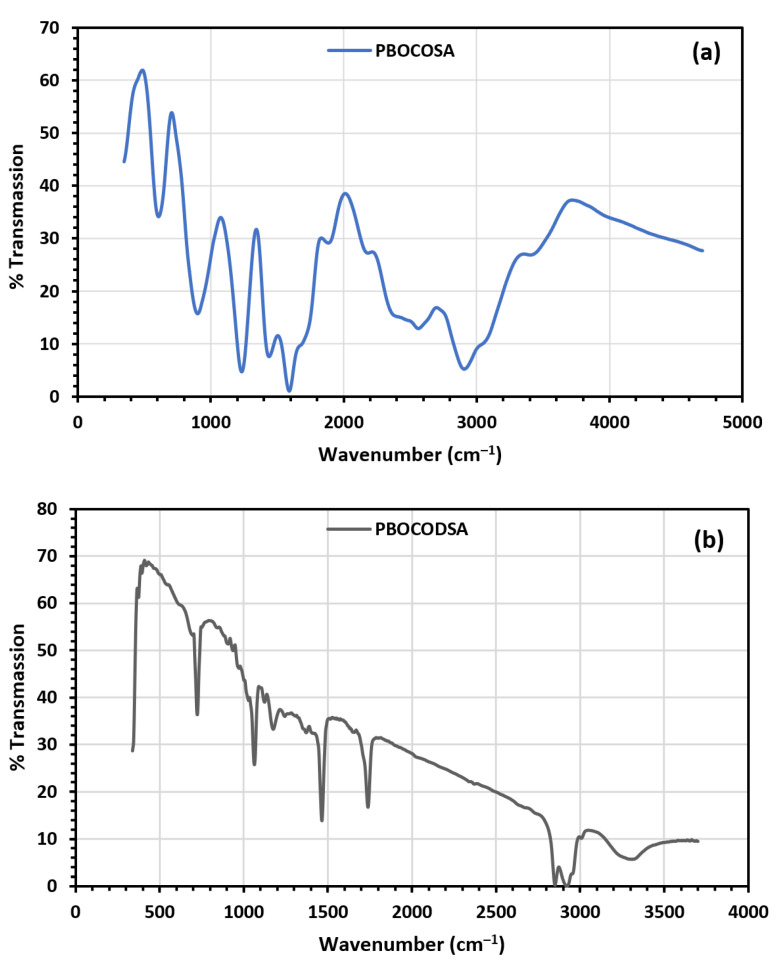
IR Spectrum of (**a**) PBOCOSA and its amided copolymer (**b**) PBOCODSA.

By using IR spectroscopy, the copolymer’s structure was verified. Figure 1 shows the IR spectra of PBOCOSA and PBOCODSA. Such a spectra displayed absorption bands at 607.6, 906, 1246, 1425.4, and 1685.8 cm^–1^. These absorption bands can be attributed to (CH_2_)n, a broad band related to the hydrogen bond O-H out-of-plane bending vibration, C-O, CH_2_, and C=O vibrational modes, respectively. Additionally, it showed bands of absorption at 2947.3 cm^–1^ that might have been caused by the stretching vibration of the C-H bonds in the paraffinic methylene groups. By examining this figure, it was discovered that similar absorption bands are shown in Figure 1a, where the corresponding absorption bands due to the (CH_2_)n, C-O, CH_2_, and C=O vibrational modes were intensely present at 750.3, 1267.3, 1467, and 1709 cm^–1^, respectively. The production of PBOCODSA may be indicated by an increase in the adsorption intensity for such bands in PBOCOSA. The CH symmetric and CH asymmetric stretch, however, caused absorption bands to form at 2862.8 and 2920.3 cm^–1^, respectively [26,27,28].

#### 3.1.2. SEM Microscopy

**Figure 2:**
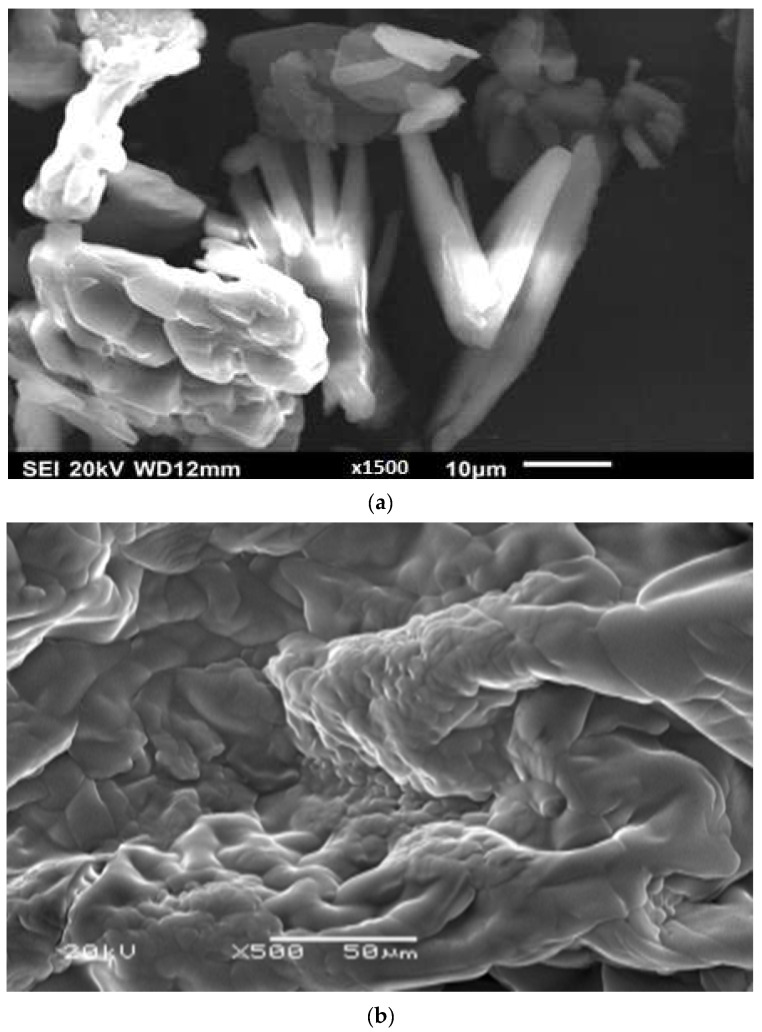
SEM micrograph of (**a**) PBOCOSA and its amided copolymer (**b**) PBOCODSA.

Figure 2 represents the SEM micrograph of both PBOCOSA and PBOCODSA. This micrograph revealed that structure of this additive is in the form of an aggregate of wrinkled copolymer chains that almost collapsed.

#### 3.1.3. XRD

**Figure 3:**
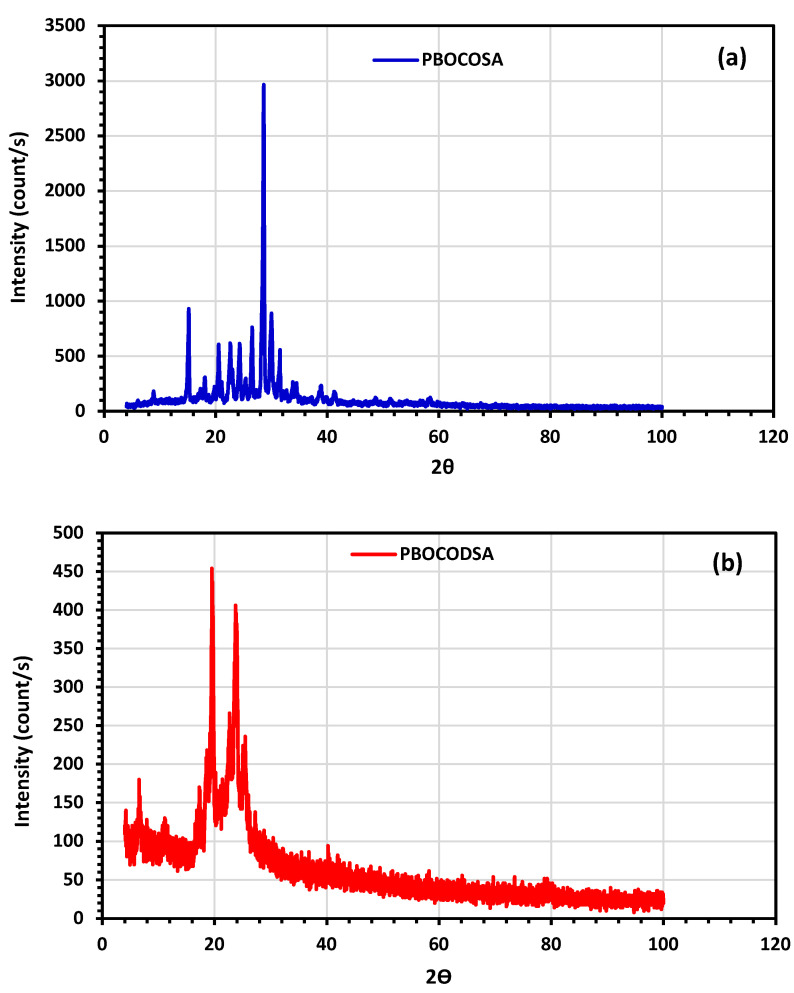
XRD of (**a**) PBOCOSA and its amided copolymer (**b**) PBOCODSA.

Patterns of XRD for polymeric additives PBOCOSA and PBOCODSA are shown in Figure 3. At 28.54°, 22.52°, 20.16°, 17.86°, and 15.06°, narrow diffraction peaks occurred, which correspond to scattering from the crystalline phase (larger crystallites) of the copolymer matrix, as shown in an XRD of PBOCOSA. Diffraction peaks of crystallites emerged at 11.93°, 13.96°, and 16.04°, with reduced relative scattering strengths, indicating considerable fluctuation in Bragg angle values in the diffractogram of PBOCODSA. PBOCOSA copolymers undergo partial structural transformation upon esterification, which may account for the observed outcome PBOCOSA. These findings show that the inclusion of such polymeric additives’ non-polar alkylated branches would hinder the stacking of the copolymer chains, resulting in lattice deformation, structural disorder, and, ultimately, a less crystalline structure than that of PBOCODSA.

#### 3.1.4. Thermogravimetric Analysis (TGA) and Stability of Copolymers

**Figure 4:**
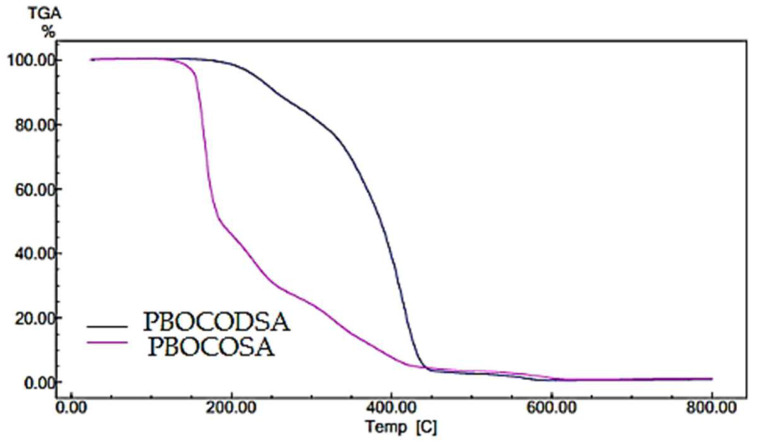
TGA thermograms of PBOCOSA and PBOCODSA.

The TGA of the polymeric products’ PBOCOSA are presented in Figure 4. It is observed from the primary thermogram that the initial decomposition of the copolymer begins at 146.32 °C, with 66.55% loss of weight after losing approximately 1.14% wt. due to water desorption. Further decomposition begins at 292.04 °C, with 29.96% loss of weight. The final decomposition begins at 445.99 °C, and beyond that, it continues leaving behind 1.340% residue, while for PBOCODSA, the initial decomposition of esterified copolymer begins at 276.08 °C, with 84% loss of weight after losing approximately 13.66% wt. due to water desorption. The final decomposition begins from 453.78 °C, and beyond that, it continues leaving behind 1.032% residue.

#### 3.1.5. Evaluation of PPD

As indicated in Table 2, a polymeric additive based on PBOCODSA having a long carbon chain (n = 18) has high potency to reduce the pour point of crude oil from 24 °C to 3 °C, i.e., the pour point depression was 21 °C. Meanwhile, the same dosage from PBOCOSA led to a depression of the pour point from 24 °C to 6 °C, i.e., pour point depression was 18 °C. The pour point values decreased gradually while increasing the concentration of this additive up to 2000 ppm, further adding improvers until reaching 3000 ppm resulted in high depression. This can be explained based on the tethering of such a polymeric additive with the paraffin wax hinders the formation of their crystals, reduces the cohesive energy, and consequently retards the growth and agglomeration of paraffin crystals [29]. This observation suggests that PBOCODSA has complex chain polymers with side chains, which conceivably provide more steric hindrance than PBOCOSA. Hence, the polymers obtained from modified oleic acid are more effective in delaying crystal growth, the crystals morphology is completely different, and there is a size reduction.

#### 3.1.6. Rheological Behaviour

**Figure 5:**
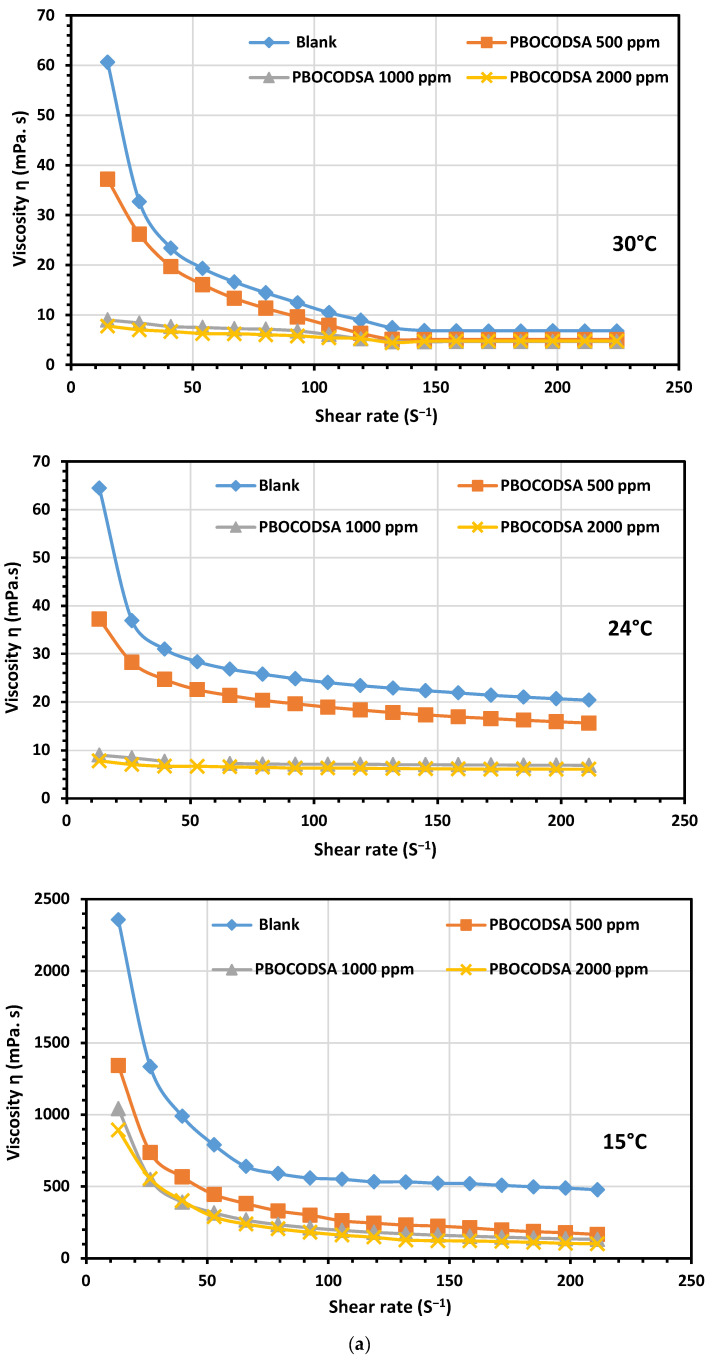

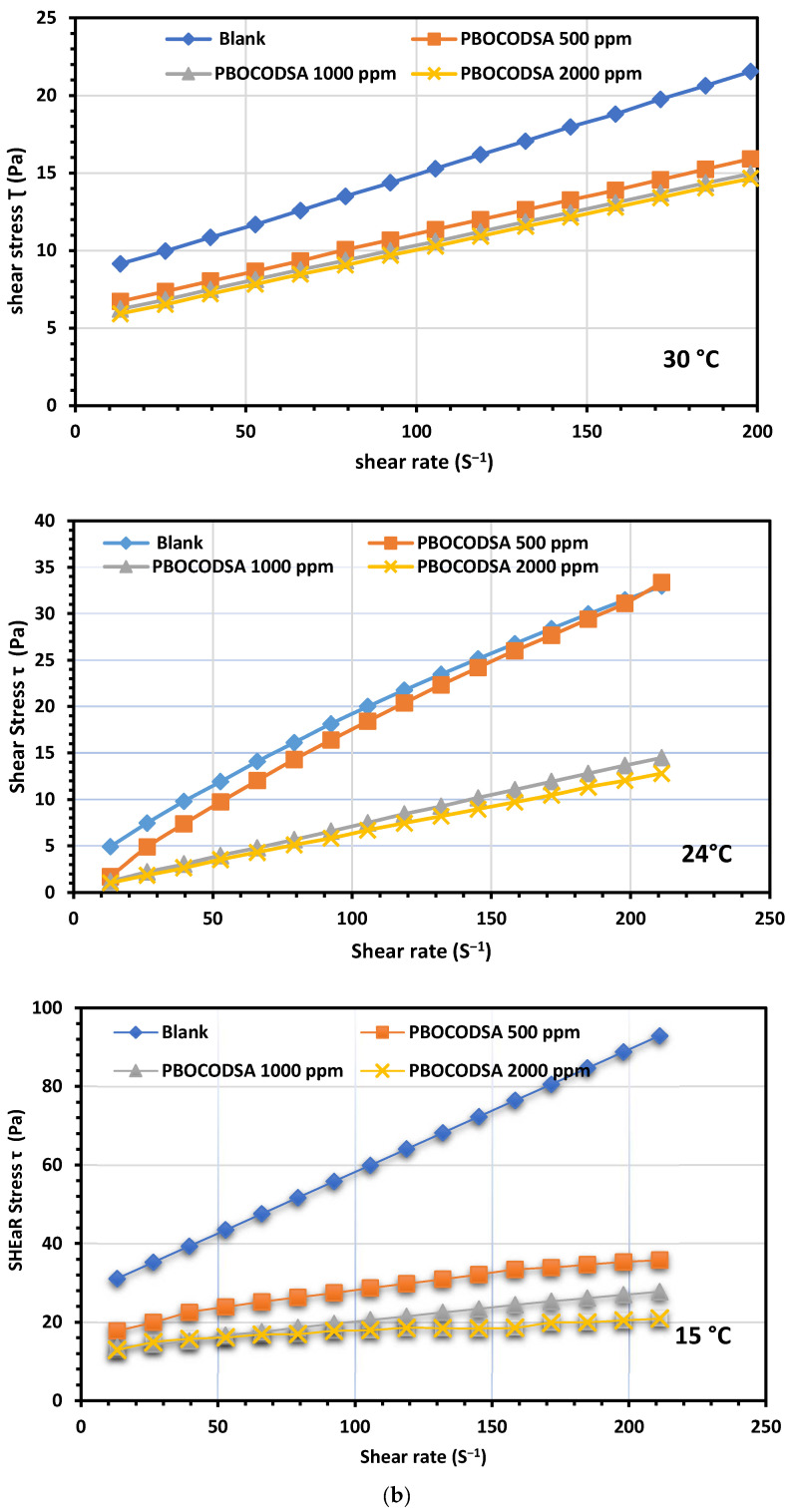
(**a**) Relation between shear rate and apparent viscosity for crude oil treated with PBOCODSA (500, 1000, and 2000ppm) at 15, 24, and 30 °C. (**b**) Relation between shear rate and shear stress for crude oil treated with PBOCODSA (500, 1000, and 2000 ppm) at 15, 24, and 30 °C.

The efficacy of the additive not only depends upon pour point measurements to describe flow properties of crude oils, but also largely on the rheological behaviours of the crude oil. The rheological behaviour is usually characterized by a shear rate–shear stress rheogram. Rheological measurements can define the direct impact of the additive on the production, transportation, and storage of crude oil. This refers to viscosity, which controls the pressure necessary to restart the flow in a pipeline or a well and corresponds to the minimum shear stress (yield value, yield point). Yield value is obtained by extrapolating the shear stress to zero shear rate on a linear plot [30]. The rheological behaviour of paraffinic waxy crude oil shifts from Newtonian to non-Newtonian when the decreasing temperature becomes lower than the pour point. Therefore, the rheological measurements were accomplished at different temperatures above and below the pour points 30, 24, and 15 °C. The findings of these measurements for plain crude oil samples and their corresponding ones treated with a PBOCODSA-based additive at different dosages (500, 1000, and 2000 ppm) are shown in Figure 5. The viscosity–shear rate relationships are shown in Figure 5a and the shear stress–shear rate relationships are recorded in Figure 5b. A waxy crude oil sample was observed to behave as Bingham plastic fluid at temperatures below its pour points; indeed, the tested petroleum crude oil (Alamin) displayed Bingham behaviour at 30 °C, which is slightly above its pour point of 24 °C, with a small yield stress of approximately 0.17 Pa. This behaved as Newtonian fluid and as the temperature reduced further, at 15 °C, it behaved more non-Newtonian, with a higher yield stress of 37.8 Pa, as shown by Figure 5a,b. This means that the crude oil could be transported with maximum energy consumption and therefore maximum pressure in the pipeline. In Table 3, it is observed that the dynamic viscosity and Bingham yield stress of the crude oil sample increases as the temperature decreases for untreated crude oil and while being treated with different dosages of PBOCODSA. In the same context, a higher decrease of the shear stress was obtained with increasing shear rate with pretreatment of crude oil with PBOCODSA. Moreover, as indicated in Table 3, the yield values for crude oil treated with PBOCODSA at dosages of 500, 1000, and 2000 ppm was lower than blank at all measurement temperatures. For yield values = 0.8 Pa at 30 °C, 0.39 Pa at 24 °C, and 20.7 Pa at 15 °C, the plastic viscosity values = 4.9 mPa.s at 30 °C, 6.71 mPa.s at 24 °C, and 80.6 mPa.s at 15 °C, with 500 ppm of PBOCODSA. For yield values = 0.07 Pa at 30 °C, 0.89 Pa at 24 °C, and 14.2 Pa at 15 °C, the plastic viscosity values = 4.73 mPa.s at 30 °C, 7.3 mPa.s at 24 °C, and 70.9 mPa.s at 15 °C, with 1000 ppm of PBOCODSA. For the minimum yield values = 0.05 Pa at 30°C, 0.35 Pa at 24°C, and 1.98 Pa at 15 °C, the plastic viscosity values = 4.33 mPa.s at 30 °C, 5.91 mPa.s at 24 °C, and 31.1 mPa.s at 15 °C, by using 2000 ppm of PBOCODSA. The remarkable efficiency of polyamides in lowering the yield shear stress value, pour point, and dynamic viscosity may be explained by the polymeric additive carrying more non-polar pendant alkyl chains. These chains better co-crystallize with non-polar groups in the long chain paraffin wax to anchor into paraffin crystals and thereby prevent the formation and growth of large wax crystals, in addition to the polar amine groups present in the polymeric backbone of this additive, which are thought to be able to form some sort of physical attraction with polar moieties of resins and asphaltenes [31]. These results are in a good correlation with the results of pour point measurements, where such a polymeric additive can promote pour point depression and simultaneously achieve a noticeable reduction in the apparent viscosity, even at low temperatures.

#### 3.1.7. Crystallization Behaviour

##### Thermal Analysis (DSC)

**Figure 6:**
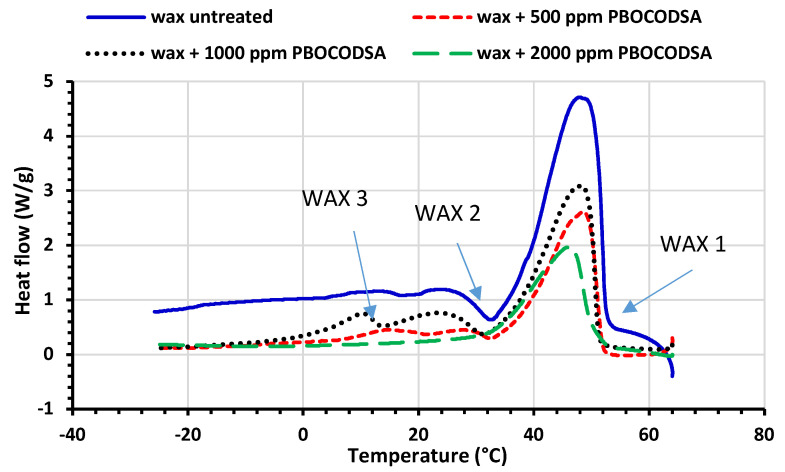
DSC thermographs of paraffin crystals in model waxy oil after 500, 1000, and 2000 ppm dosage of PBOCODSA during cooling at a rate of 10 °C/min.

This experimental technique was used to detect changes in crude oil behaviour at low temperatures due to the presence of an inhibitor. The additives tested with PBOCODSA were added in different concentrations (500, 1000, and 2000 ppm) to the studied crude oils. The exotherms (Figure 6) can be due to either solid–solid (orthorhombic to hexagonal) transition or solid–liquid melting transition. WAT reduces with an increasing equilibration time. The presence of three WATs is due to the presence of more than one type of paraffin differing in thermal properties. The enthalpy change of wax crystallization in the crude oil was measured through the DSC cooling curves of untreated and treated paraffin. The crystallization enthalpy was used for the determination of the effect of precipitated wax versus temperature. The value for virgin paraffin was 86.97 J/g and that of the treated paraffin with PBOCODSA was 64.18 J/g, with 500 ppm. This observation means that the PPD has a great effect on the total amount of wax that would completely precipitate. The DSC cooling curves of untreated and treated paraffin with the PBOCODSA polymers’ additive at concentration of 1000 ppm have broadened and shifted to lower temperatures. According to these findings, crystallization slowed down and took place at lower temperatures. The temperature at which the heat flow changed during cooling represents the onset temperature (T*onset*) of crystallization. T*onset* in relation to additives for the waxy oils at a cooling rate of 10 °C /min is summarized in Table 4. Moreover, T*onset* further decreased when PBOCODSA was used; it decreased with the addition of 2000 ppm, demonstrating the effect of the content of mixture from polymers elevated from the presence of stearyl amine. The area of the peak displays a declining tendency, which means that crystallizing processes have occurred when the mixture was continually cooling.

##### XRD

**Figure 7:**
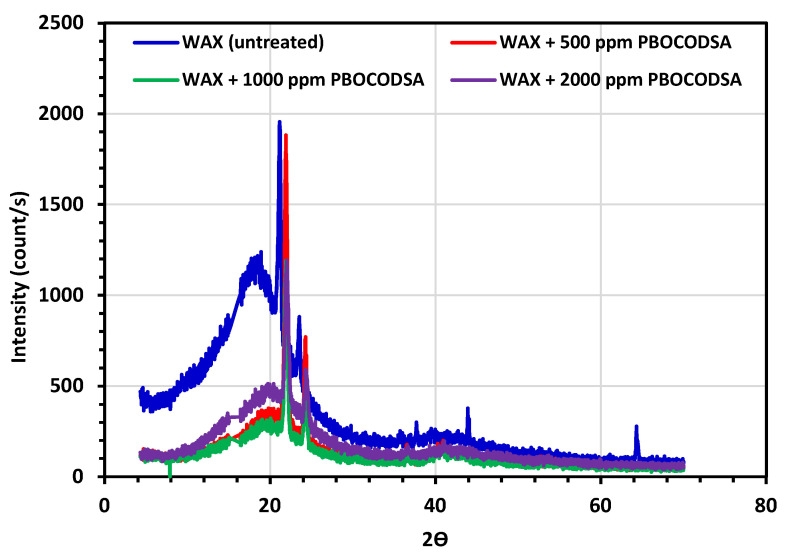
XRD of untreated paraffin wax from crude oil and its corresponding treatment with PBOCODSA.

Figure 7 represents the XRD of untreated paraffin wax and treatment with PBOCODSA. These XRD grams showed that the peaks due to crystalline structure appeared at 2θ ≈ 21.0068 and 23.37 for both untreated wax and its counterpart treated by PBOCODSA. Moreover, XRD patterns showed that the degree of crystallinity of the untreated wax is higher than that for the paraffin wax treated by PBOCODSA at dosages of 500, 1000, and 2000 ppm.

##### Microscopic Observations of Wax Structure Modification

**Figure 8:**
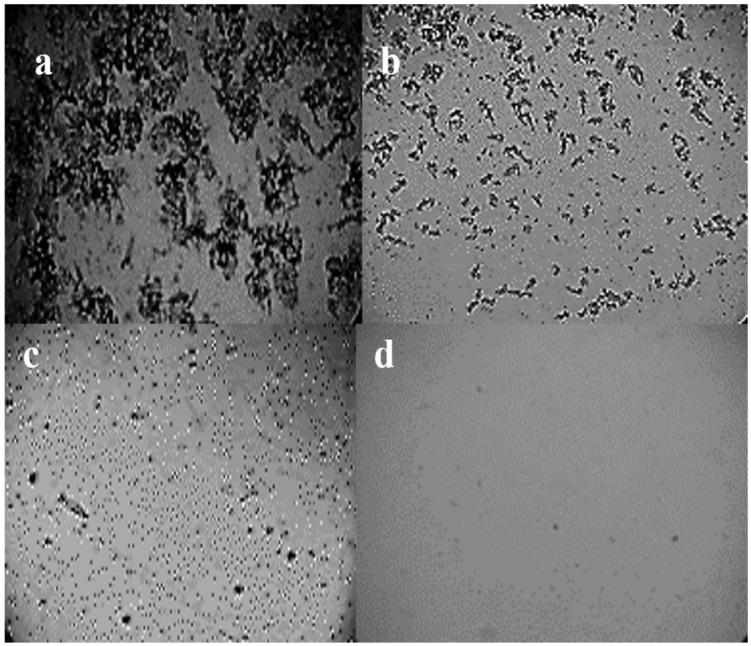
Photographs of paraffin crystals from waxy crude oil untreated (**a**) and treated with PBOCODSA (500 (**b**), 1000 (**c**),and 2000 (**d**) ppm).

In the absence of additive for virgin crude oil, there was a dense amount of small, dark particles of wax crystals where aggregates had haphazard arrangements (Figure 8a). When the crude oil was treated with 500 ppm PBOCODSA, it is notable that the crystals were dramatically changed to tiny particles that were treated with PBOCODSA, with the wax-crystals aggregating into large flocs. Further additive, with a concentration of 1000 ppm, resulted in a decrease in crystals and a clear sample from crystals when treated with PBOCODSA, where the structure of the wax-crystal flocs is loose (Figure 8d). A reduction of the crystals’ size was found by the further addition of improvers PBOCODSA with a concentration of 2000 ppm. In comparison with Al-Sabagh [20], who investigated the copolymer aliphatic side chain styrene maleic anhydride co-amide which exhibited the maximum pour point depression (ΔPP = 21 °C at 1500 ppm), our PBOCODSA has the same depression result but at 2000 ppm. From the results, with the interaction mechanism under low temperature and without PBOCODSA addition, the high content of the wax crystals with short distances facilitates hydrophobic bonds that form wax aggregates, as shown in Figure 8a. These convert the waxy crude oil to a two-phase system: the lower phase is wax and the upper phase is oil. Moreover, wax aggregates can be grown due to the interaction of the wax intermolecules, forming networks and hampering the crude oil flowability. These results are consistent with the high pour point of waxy crude oil at 24 °C. By using PBOCODSA with polar and non-polar groups, the interaction mechanism refers to adsorption or co-crystallization or both [30,32,33]. The co-crystallization occurs between non-polar alkyl groups of PBOCODSA molecules and wax molecules. Meanwhile PBOCODSA is composed of polar amine groups in addition to the non-polar groups. These polar groups exist on the surface of the wax crystals and absorb low molecular weight polar substances to the wax-crystal surfaces and form a layer of energy barrier, called a solvated layer [34]. These solvated layers not only change the interface properties between the wax crystals and the oil phase, but they also prevent the connection of the wax crystals and the formation of larger crystal aggregates [35,36].

## 4. Conclusions

In this study, the production of commodity comb-like PBOCODSA amided using stearyl amine having a long carbon chain (n = 18) meets the demands of green chemistry by using renewable resources; they were successfully synthesized by amidation of PBOCOSA with alkyl amine (streaylamine) to form a branched copolymer. The long side chains of PPDs are similar to the wax crystals, with this part functioning by providing nucleation sites and so enabling the bulk stream to remain pumpable, pourable, and filterable. PBOCODSA’s potential feasibility as PPDs was investigated by studying its impact on the crystallization of paraffin wax and cold flowability of petroleum waxy crude oil. The findings of DSC, XRD, and LM showed that PBOCODSA reduces the paraffin crystallization temperature and enthalpies that lead to inhibit the formation of the layer structure of paraffin wax and intensify the growth of microscopic paraffin crystals. The maximum pour point depression of the crude oil (∆PP) occurred at 21 °C and was achieved by using PPD at 2000 ppm. This depression was associated with decreasing the paraffin crystal size and suppressing the formation of crystal platelets. PBOCODSA has the characteristics to be an efficient paraffin crystal modifier, including the long hydrocarbon chains derived from the alkyl side chain and the hydrophilic part (amide groups). The function of PBOCODSA has been to change the nature of the paraffin crystals and to destroy cohesive forces between the crystals, thereby reducing the risk of forming three-dimensional networks.

## Figures and Tables

**Table 1:** Physical characteristics of Alamin crude oil [1].

Physicochemical Parameters	Values	Method
Density (g/cm^3^)	0.8180	ASTM D1298
API Gravity	41.38	ASTM D1298
Kinematic Viscosity (c. st.)		ASTM D445
60 °C	7.43	
100 °C	3.67	
Pour Point, PP (°C)	24	ASTM D97
Wax Content (%)	12.40	UOP46
Wax Appearance Temperature (WAT1) (°C)	53.23	
Asphaltene Content (%)	1.32	ASTM D6560
Water Content (% vol.)	0.10	ASTMD-4006
Sediment (% m/m)	0.034	ASTMD-473
Water and Sediment (% vol)	0.15	ASTMD-4007
Salt Content (PT B)	85	ASTMD-3230
Ash Content (% wt)	0.018	ASTMD-482

**Table 2:** Effect of concentration of polymeric additives on the pour point of crude oil.

Additive	Pour Point (°C) of Crude Oil by Using Different Additive Concentrations (ppm)					Pour Point Depression (°C)
	Blank	500	1000	2000	3000	
PBOCOSA	24	21	12	6	6	18
PBOCODSA	24	15	9	3	0	24

**Table 3:** Rheological properties of Egyptian heavy crude oil.

	Flow Improvers	Temperature	Plastic Viscosity	Yield Value
Blank		30	6.64	0.17
		24	18.5	2.62
		15	370	37.8
PBOCODSA	500 ppm	30	4.9	0.8
		24	6.71	0.39
		15	80.6	20.7
	1000 ppm	30	4.73	0.07
		24	7.3	0.89
		15	70.9	14.2
	2000 ppm	30	4.33	0.1
		24	5.91	0.35
		15	31.1	1.98

**Table 4:** T*onset* for crystallization of crude oil with and without improvers at a cooling rate of 10 °C/min.

Copolymeric Additive		Wax Appearance Temperature (WAT1) (°C)	Solid–Solid Phase Transition Temperature (°C)	∆H_crystallization_ (J/g)
0		54.23	40.86	86.97
PBOCODSA	500 ppm	52.02	38.65	64.18
	1000 ppm	51.79	34.76	64.07
	2000 ppm	50.23	27.19	37.35

## Data Availability

Not applicable.
